# Author Correction: Hyperbolic exciton polaritons in a van der Waals magnet

**DOI:** 10.1038/s41467-024-45809-8

**Published:** 2024-02-27

**Authors:** Francesco L. Ruta, Shuai Zhang, Yinming Shao, Samuel L. Moore, Swagata Acharya, Zhiyuan Sun, Siyuan Qiu, Johannes Geurs, Brian S. Y. Kim, Matthew Fu, Daniel G. Chica, Dimitar Pashov, Xiaodong Xu, Di Xiao, Milan Delor, X-Y. Zhu, Andrew J. Millis, Xavier Roy, James C. Hone, Cory R. Dean, Mikhail I. Katsnelson, Mark van Schilfgaarde, D. N. Basov

**Affiliations:** 1 Department of Physics, Columbia University, New York, NY USA; 2 Department of Applied Physics and Applied Mathematics, Columbia University, New York, NY USA; 3 National Renewable Energy Laboratory, Golden, CO USA; 4 Columbia Nano Initiative, Columbia University, New York, NY USA; 5 Department of Mechanical Engineering, Columbia University, New York, NY USA; 6 Department of Chemistry, Columbia University, New York, NY USA; 7 Theory and Simulation of Condensed Matter, King’s College London, London, UK; 8 Department of Physics, University of Washington, Seattle, WA USA; 9 Department of Materials Science and Engineering, University of Washington, Seattle, WA USA; 10 Center for Computational Quantum Physics, Flatiron Institute, New York, NY USA; 11 Institute for Molecules and Materials, Radboud University, Nijmegen, Netherlands

**Keywords:** Polaritons, Sub-wavelength optics, Magnetic properties and materials, Two-dimensional materials

Correction to: *Nature Communications* 10.1038/s41467-023-44100-6, published online 13 December 2023

The original version of this Article contained an error in the reference list order. References 47 and 48 were erroneously switched with references 49 and 50, respectively. This has been corrected in the PDF and HTML versions of the Article.

