# Supplementary material for: Hyperbolic exciton polaritons in a van der Waals magnet
Source: Nat Commun. 2023 Dec 13;14:8261. doi: 10.1038/s41467-023-44100-6 (PMC10716151; doi:10.1038/s41467-023-44100-6)
Supplement: Supplementary file 1 — Supplementary Information [file 41467_2023_44100_MOESM1_ESM.pdf]

Supplementary Information for

Hyperbolic exciton polaritons in a van der Waals magnet

Francesco L. Ruta, Shuai Zhang*, Yinming Shao, Samuel L. Moore, Swagata Acharya, Zhiyuan Sun, Siyuan Qiu, Johannes Geurs, Brian S. Y. Kim, Matthew Fu, Daniel G. Chica, Dimitar Pashov, Xiaodong Xu, Di Xiao, Milan Delor, X-Y. Zhu, Andrew J. Millis, Xavier Roy, James C. Hone, Cory R. Dean, Mikhail I. Katsnelson, Mark van Schilfgaarde, D. N. Basov**

Correspond to: *f.ruta@columbia.edu, *sz2822@columbia.edu, *db3056@columbia.edu

Table of Contents **pg(s)**

Supplementary Figures

1. *Far-field infrared spectroscopy with b-axis polarization* 1
2. *Far-field infrared spectroscopy with a-axis polarization* 2
3. *Fitting c-axis dielectric constant to near-field data* 2
4. *Im r_p in complex-k space* 5
5. *Hyperbolic surface polaritons* 7
6. *Rabi splitting in complex-ω electrodynamics* 9
7. *Fourier analysis of near-field amplitude data* 10
8. *Geometrical corrections to the in-plane wavevector* 11
9. *Temperature and thickness dependence of hyperbolic exciton polaritons* 12
10. *Atomic force microscopy of CrSBr microcrystals* 12
11. *Temperature dependence of exciton spectral weight* 13

Supplementary Tables

1. *Lorentz and Tauc-Lorentz oscillators initial model parameters* 1

Supplementary Notes

1. Hyperbolic polaritons near the light cone

- I. *Reflection from biaxial slab* 3-4
- II. *Note on maxima of Im r_p* 4
- III. *Hyperbolic polariton modes* 5-6

2. Coupled excitons and waveguide modes 8

3. Geometrical correction to the in-plane wavevector 11

Supplementary References 14

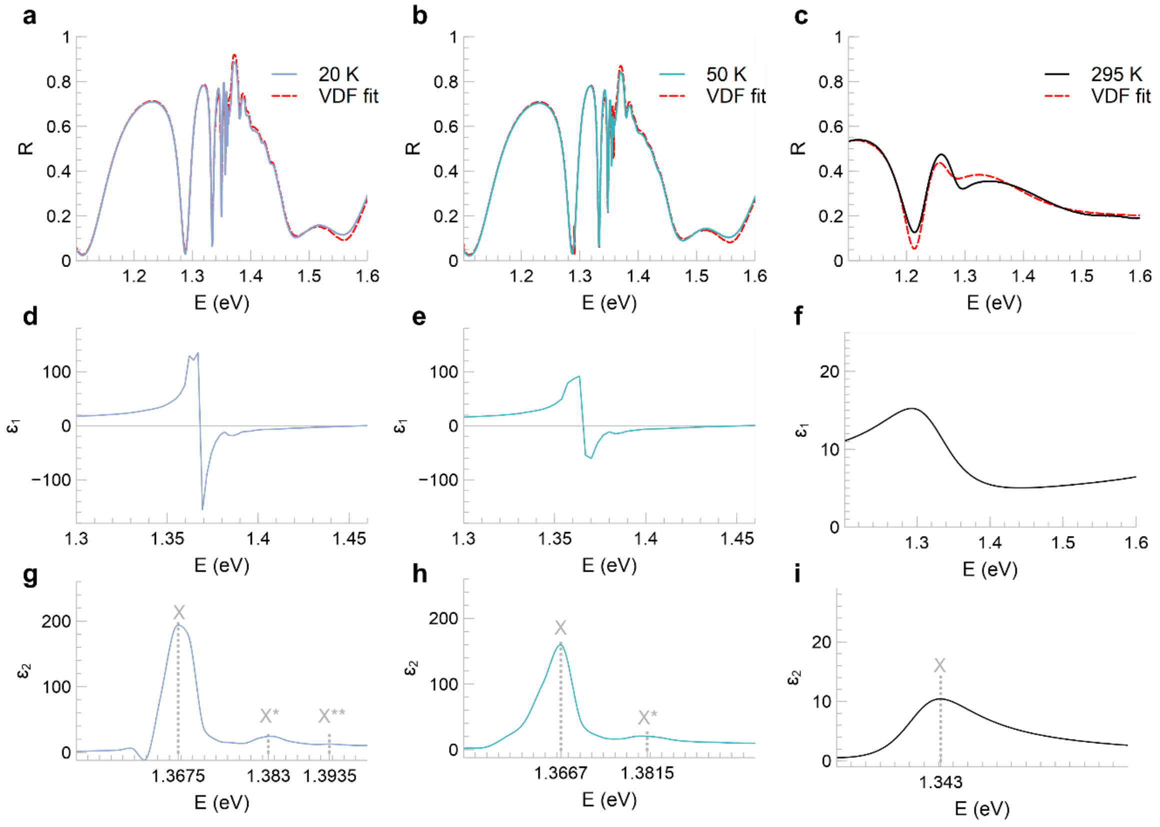

Supplementary Figure 1: Far-field infrared spectroscopy with b-axis polarization. Far-field reflectance spectra from Fourier transform infrared spectroscopy along the b-axis for, **a**, 20 K, **b**, 50 K, and **c**, 295 K. **d-f,** corresponding real dielectric functions (ε_1) obtained from variational dielectric function (VDF) fitting. Linewidths are orders of magnitude lower at cryogenic temperatures, and **d** has a larger oscillator strength than **e** (Supplementary Table 1). **g-i**, corresponding imaginary dielectric functions (ε_2) obtained from VDF fitting (solid lines). Three peaks in **g** are the exciton (X) at $E = 1.3675$ eV and two sidebands (X*, X**) at $E = 1.383$ eV and 1.3935 eV, respectively. X and X* are observed in **h** at $E =$ 1.3667 eV and 1.3815 eV, respectively. Only X is observed in **i** peaked at $E = 1.343$ eV.

Supplementary Table 1: Lorentz and Tauc-Lorentz oscillators initial model parameters.

T (K)	Type	ε_∞ | $\omega_{g,i}$ (cm^{-1})	ω_0 | ω_i (cm^{-1})	*f* (cm^{-2}) | A_i (cm^{-1})	γ | γ_i (cm^{-1})
295	Lorentz	3.41	9349.4	12391104	2218.8
	Tauc-Lorentz	9559.56	10637.25	855097.98	982.25
	Tauc-Lorentz	12979.33	11656.99	6963112.86	1480.71
50	Lorentz	3.1	11307.76	22188621.83	341.91
	Tauc-Lorentz	10689.97	11007.05	9443575.64	46.06
	Tauc-Lorentz	13456.81	14135.51	2381064.29	495.51
	Tauc-Lorentz	15824.97	13875.94	10633893.2	1142.09
20	Lorentz	2.67	11316.49	28553206	373.14
	Tauc-Lorentz	10779.76	11024.22	17284378.15	28.97
	Tauc-Lorentz	13493.87	14185.4	2701528.49	458.6
	Tauc-Lorentz	15734.46	13329.45	9155736.47	2047.61

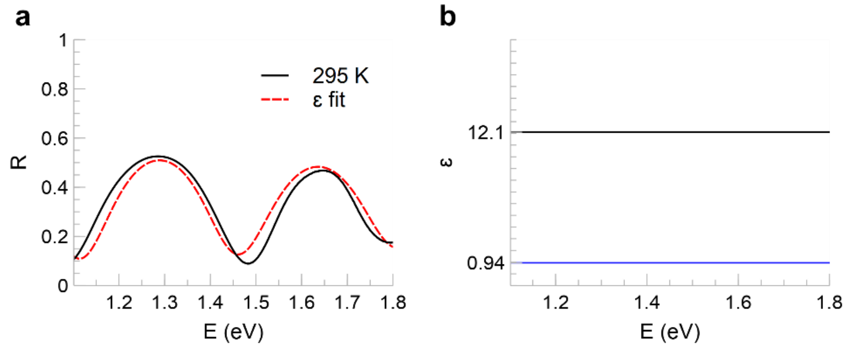

Supplementary Figure 2: Far-field infrared spectroscopy with a-axis polarization. **a**, Far-field reflectance spectrum from Fourier transform infrared spectroscopy polarized along the CrSBr $\underline{a}$-axis at room temperature (black). Dielectric function fit (red dashed line) using a complex dielectric constant. **b**, extracted $\underline{a}$-axis dielectric constant is $\varepsilon_a = 12.41 + 0.94i$.

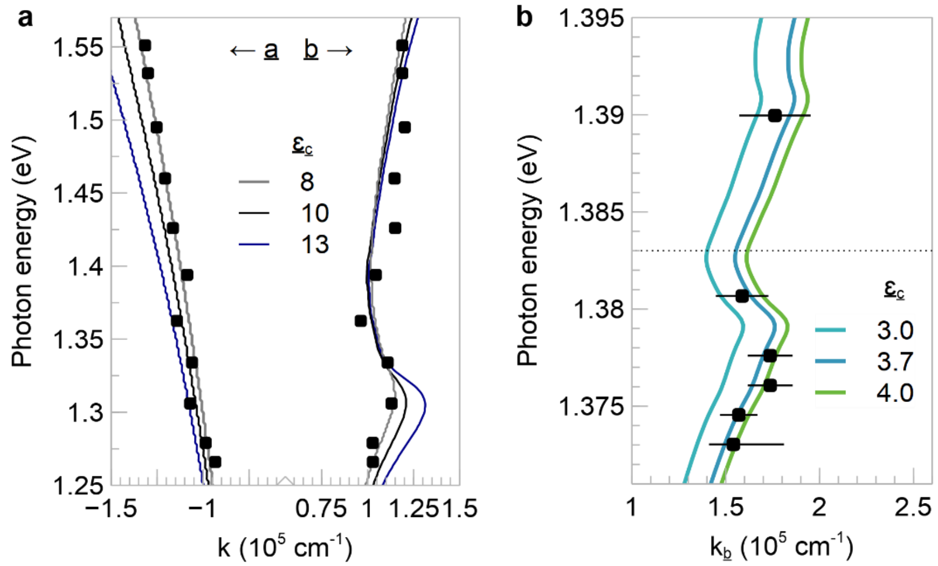

Supplementary Figure 3: Fitting c-axis dielectric constant to near-field data. **a**, waveguide mode dispersions for various values of ε_c, the out-of-plane dielectric constant. $\varepsilon_c = 8$ is the best-fit value for room-temperature $\underline{a}$-axis near-field data (black squares, $k < 0$). $\varepsilon_c = 8$ also fits $\underline{b}$-axis near-field data ($k > 0$) well, but these data were not considered during least-squares fitting. **b**, hyperbolic exciton polariton dispersions for various values of ε_c. $\varepsilon_c = 3.7$ is the best-fit value for 20 K near-field data on a 107 nm sample (black squares). For 50 K, we use the waveguide mode in Figure 3f to extract $\varepsilon_c = 4.5$.

Supplementary Note 1: Hyperbolic polaritons near the light cone

I. Reflection from biaxial slab

CrSBr is orthorhombic and thus biaxial. For calculating the complex reflection coefficients of p- and s-polarized light, $r_p(\omega, k)$ and $r_s(\omega, k)$, respectively, at arbitrary orientations, we employ a general 4×4 propagation matrix formalism[1-2]. The CrSBr dielectric tensor $\overleftrightarrow{\varepsilon}$ can be written for any arbitrary orientation by rotating its basis. In this work, we only rotate the basis in-plane (to compute isofrequency contours in Figure 4a) and so only one parameter ϕ is necessary:

$$\overleftrightarrow{\varepsilon}'(\omega) = R^{-1}(\phi)\overleftrightarrow{\varepsilon}(\omega)R(\phi) \qquad (S1)$$

$$R = \begin{bmatrix} \cos\phi & \sin\phi & 0 \\ -\sin\phi & \cos\phi & 0 \\ 0 & 0 & 1 \end{bmatrix} \qquad (S2)$$

Maxwell's curl equations in an arbitrary anisotropic medium can be written in the form of a first-order system of differential equations (in SI units):

$$\frac{\partial \Psi}{\partial z'} = i\Delta\Psi \qquad (S3)$$

where $\Psi = \left[\sqrt{\varepsilon_0}E_x \quad \sqrt{\mu_0}H_y \quad \sqrt{\varepsilon_0}E_y \quad \sqrt{\mu_0}H_x\right]^T$ and the components of the Δ-matrix are as follows when the *xz* plane is the plane of incidence:

$$\Delta = \frac{\omega}{c}\begin{bmatrix} -\varepsilon'_{zx}\mathrm{kc}/\omega\varepsilon'_{zz} & 1 - \mathrm{k}^2\mathrm{c}^2/\omega^2\varepsilon'_{zz} & -\varepsilon'_{zy}\mathrm{kc}/\omega\varepsilon'_{zz} & 0 \\ \varepsilon'_{xx} - \varepsilon'_{xz}\varepsilon'_{zx}/\varepsilon'_{zz} & -\varepsilon'_{xz}\mathrm{kc}/\omega\varepsilon'_{zz} & \varepsilon'_{xy} - \varepsilon'_{xz}\varepsilon'_{zy}/\varepsilon'_{zz} & 0 \\ 0 & 0 & 0 & 1 \\ \varepsilon'_{yx} - \varepsilon'_{yz}\varepsilon'_{zx}/\varepsilon'_{zz} & \varepsilon'_{yz}\mathrm{kc}/\omega\varepsilon'_{zz} & \varepsilon'_{yy} - \mathrm{k}^2\mathrm{c}^2/\omega^2 - \varepsilon'_{yz}\varepsilon'_{zy}/\varepsilon'_{zz} & 0 \end{bmatrix} \qquad (S4)$$

where k and ω are the in-plane momentum and frequency of light, respectively. The general solution to the system of differential equations in Equation S3 that gives the electromagnetic field components after traveling a distance d inside the medium is:

$$\Psi(z' + d) = e^{i\Delta d}\Psi(z') \qquad (S5)$$

The matrix $P(d) = e^{i\Delta d}$ is called the propagation matrix. To evaluate the exponential of Δ, we compute its eigenvalues $\{k_{zi}\}$ and eigenvectors V.

$$P(d) = V \begin{bmatrix} e^{ik_{z1}d} & 0 & 0 & 0 \\ 0 & e^{ik_{z2}d} & 0 & 0 \\ 0 & 0 & e^{ik_{z3}d} & 0 \\ 0 & 0 & 0 & e^{ik_{z4}d} \end{bmatrix} V^{-1} \tag{S6}$$

Finally, $r_p(\omega, k)$ and $r_s(\omega, k)$ can be calculated from the continuity requirements at the interfaces. The incident and exit media have isotropic refractive indices of $n_i = 1$ and n_t, respectively.

$$r_p(\omega, k) = \frac{A_+D_- - B_-C_+}{B_-C_- - A_-D_-}, \quad r_s(\omega, k) = \frac{A_-D_+ - B_+C_-}{B_-C_- - A_-D_-} \tag{S7}$$

where:

$$A_{+,-} = n_t \mathrm{P}_{12} - \cos\gamma_\mathrm{t}\,\mathrm{P}_{22} \pm \cos\gamma_\mathrm{i}\,(\mathrm{n_t P_{11}} - \cos\gamma_\mathrm{t}\,\mathrm{P}_{21})$$
$$B_{+,-} = (\mathrm{n_t P_{13}} - \cos\gamma_\mathrm{t}\,\mathrm{P}_{23}) \pm \cos\gamma_\mathrm{i}\,(\mathrm{n_t P_{14}} - \cos\gamma_\mathrm{t}\,\mathrm{P}_{24})$$
$$C_{+,-} = (\mathrm{n_t}\cos\gamma_\mathrm{t}\,\mathrm{P}_{32} - \mathrm{P}_{42}) \pm \cos\gamma_\mathrm{i}\,(\mathrm{n_t}\cos\gamma_\mathrm{t}\,\mathrm{P}_{31} - \mathrm{P}_{41})$$
$$D_{+,-} = (\mathrm{n_t}\cos\gamma_\mathrm{t}\,\mathrm{P}_{33} - \mathrm{P}_{43}) \pm \cos\gamma_\mathrm{i}\,(\mathrm{n_t}\cos\gamma_\mathrm{t}\,\mathrm{P}_{34} - \mathrm{P}_{44})$$

and $\cos\gamma_{\mathrm{i,t}} = \sqrt{1 - \left(kc/n_{i,t}\omega\right)^2}$ are cosines of the angles of incidence and transmission, accordingly. $r_s(\omega, k)$ will be used to calculate TE_0 mode momenta in Supplementary Figure 9.

II. Note on maxima of $\mathrm{Im}\, r_p$

When dissipation ($\mathrm{Im}\, k$) is large, as in Figure 4c, $\max \mathrm{Im}\, r_p$ may no longer be a good indicator of poles. Consider $r_p(k)$ at fixed ω that is non-holomorphic at $k = k_p$ but analytic on $0 < |k - k_p| < z$ with Laurent series:

$$r_p(k) = \sum_{n=1}^{\infty} \frac{b_n}{\left(k - k_p\right)^n} + \sum_{m=0}^{\infty} a_m \left(k - k_p\right)^m \tag{S8}$$

We plot $\mathrm{Im}\, r_p$ from Figure 4c in complex-k space with true dissipation (Supplementary Figure 4a) and reduced dissipation (Supplementary Figure 4b). Note in Supplementary Figure 4b, the pole resembles a simple pole with real coefficient (*i.e.* $\mathrm{Im}\, b_1 \approx 0$ and $b_{n>1} \approx 0$, inset of Supplementary Figure 4b), such that $\mathrm{argmax}\, \mathrm{Im}\, r_p(k) \approx k_p$. When larger losses are introduced, k_p begins to deviate from a simple pole with real coefficient (Supplementary Figure 4a). Either $\mathrm{Im}\, b_1$ or $b_{n>1}$ are now non-negligible. Thus, the usual $\mathrm{Im}\, r_p$ heuristics for poles are invalid. For this reason, computed dispersions may not necessarily align with maxima of $\mathrm{Im}\, r_p$ in all figures.

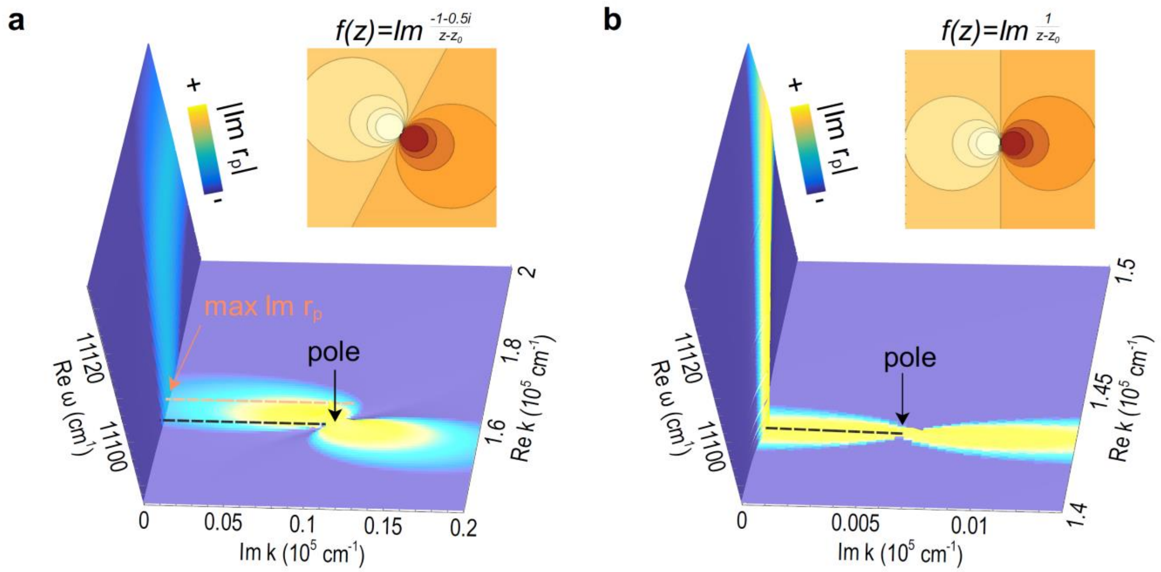

Supplementary Figure 4: Im $\mathbf{r_P}$ in complex-k space. Imaginary part of the p-polarized reflection coefficient r_p in complex-k space at fixed photon energy $E = 1.375$ eV with **a**, experimental and, **b**, artificially reduced dissipation. $\max \mathrm{Im}\, r_p$ does not align with the complex pole of r_p when dissipation is large in **a**. With reduced dissipation in **b**, however, the pole resembles a simple pole with real coefficient (inset) at approximately $\max \mathrm{Im}\, r_p$.

III. Hyperbolic polariton modes

When we consider an orientation where the CrSBr dielectric tensor $\overleftrightarrow{\varepsilon}$ is diagonal, *e.g.* when the principal *b*- and *c*-axes are aligned to the *xz* axes, r_p simplifies to a familiar form:

$$r_p(\omega, k) = \frac{r_{01} + r_{12} e^{2ik_z d}}{1 - r_{01} r_{21} e^{2ik_z d}} \qquad (S9)$$

where:

$$r_{uv} = \frac{Q_v - Q_u}{Q_v + Q_u}, \quad Q_u = \frac{\varepsilon_x^u}{k_z^u}, \text{ and } k_z = \sqrt{\frac{\varepsilon_x}{\varepsilon_z}(k_m^2 - k^2)}.$$

d is the thickness of the slab and $k_0 = \omega/c$ is the momentum of vacuum photons. Now consider a geometry where substrate and superstrate are both vacuum. Equation S9 further simplifies and we get a simple condition for the polariton modes of the slab:

$$r_{01}^2 = e^{-2ik_z d}, \qquad r_{01} = \frac{\sqrt{k_0^2 - k^2} \mp \sqrt{\frac{1}{\varepsilon_x \varepsilon_z}(k_m^2 - k^2)}}{\sqrt{k_0^2 - k^2} \pm \sqrt{\frac{1}{\varepsilon_x \varepsilon_z}(k_m^2 - k^2)}} \qquad (S10)$$

We discuss the hyperbolic case where $\varepsilon_x \varepsilon_z < 0$. We observe that the fundamental hyperbolic mode is a surface mode when its dispersion is between the vacuum light cone k_0 and material light cone $k_m = \sqrt{\varepsilon_z} k_0$ by the following argument. Neglecting dissipation ($\mathrm{Im}\, k = 0$), one has

$$\begin{aligned} \mathrm{Im}\, r_{01} = 0 \quad & k_0 \le k \le k_m \\ \mathrm{Im}\, r_{01} \ne 0 \quad & \quad k > k_m \end{aligned} \tag{S11}$$

Therefore, to satisfy Equation S10 as k varies in the range $k_0 \le k \le k_m$, k_z must be purely imaginary since r_{01}^2 is purely real. That is to say, the polariton is a surface mode. When $k > k_m$, the solution for k_z becomes complex since r_{01} can in general be complex, meaning the mode will have some oscillatory out-of-plane component like a typical bulk hyperbolic polariton. Furthermore, expanding the positive branch of r_{01}^2 and $e^{-i2k_z d}$ about k_m:

$$r_{01}^2 \approx 1 + 4\sqrt{\frac{k_m^2 - k^2}{(-\varepsilon_x\varepsilon_z)(k^2 - k_0^2)}}, \qquad e^{-i2k_z d} \approx 1 + 2d\sqrt{\frac{-\varepsilon_x}{\varepsilon_z}(k_m^2 - k^2)} \tag{S12}$$

As we move slightly left to $k < k_m$, we see that both r_{01}^2 and $e^{-i2k_z d}$ are increasing from one since $-\varepsilon_x\varepsilon_z$ and $-\varepsilon_x/\varepsilon_z > 0$. Moreover, $r_{01}^2 \to +\infty$ as $k \to \sqrt{(1 - 1/\varepsilon_x)\,/(1 - 1/\varepsilon_x\varepsilon_z\,)}k_0 \equiv k^*$ and clearly $k_0 < k^* < k_m$. Thus, as we move farther below k_m, r_{01}^2 must increase rapidly while $e^{-i2k_z d}$ grows comparatively slowly. If the lefthand slope of r_{01}^2 is smaller than that of $e^{-i2k_z d}$ at k_m, then these functions must intersect at some point (Supplementary Figure 5b). By comparing derivatives of Equation S10, we can solve for the frequency range where this is true:

$$|\varepsilon_x(\omega)| \ge \left(\pi\omega d\sqrt{\varepsilon_z - 1}\right)^{-1} \tag{S13}$$

The range of frequencies satisfying Equation S13 admit a solution to Equation S10 for $k^* < k < k_m$, and by the arguments following Equation S11 it must be a surface mode. Saturation of the bound in Equation S13 corresponds to crossing of the material light cone. When Equation S13 is not satisfied, the fundamental mode still exists, but as a bulk mode at $k > k_m$.

Suppose now the dielectric function is a single Lorentz oscillator (Equation M1) with $\gamma = 0$. Then $\varepsilon_x \to -\infty$ as $\omega \to \omega_0$ from above. So $k^* \to k_0$ and $e^{-i2k_z d}$ diverges immediately below k_m, and we have $k(\omega_0) \to k_0$ for the surface mode as illustrated in Supplementary Figure 5a. Meanwhile, higher-order modes with $k > k_m$ always have $r_{01}^2 \to 1$ as $\omega \to \omega_0$ and the self-oscillation condition is simply $n\pi/d = \sqrt{-\varepsilon_x(k^2 - k_m^2)/\varepsilon_z}$. The righthand side will always diverge unless $k = k_m$ for all mode orders n, thus forcing $k(\omega_0) \to k_m$.

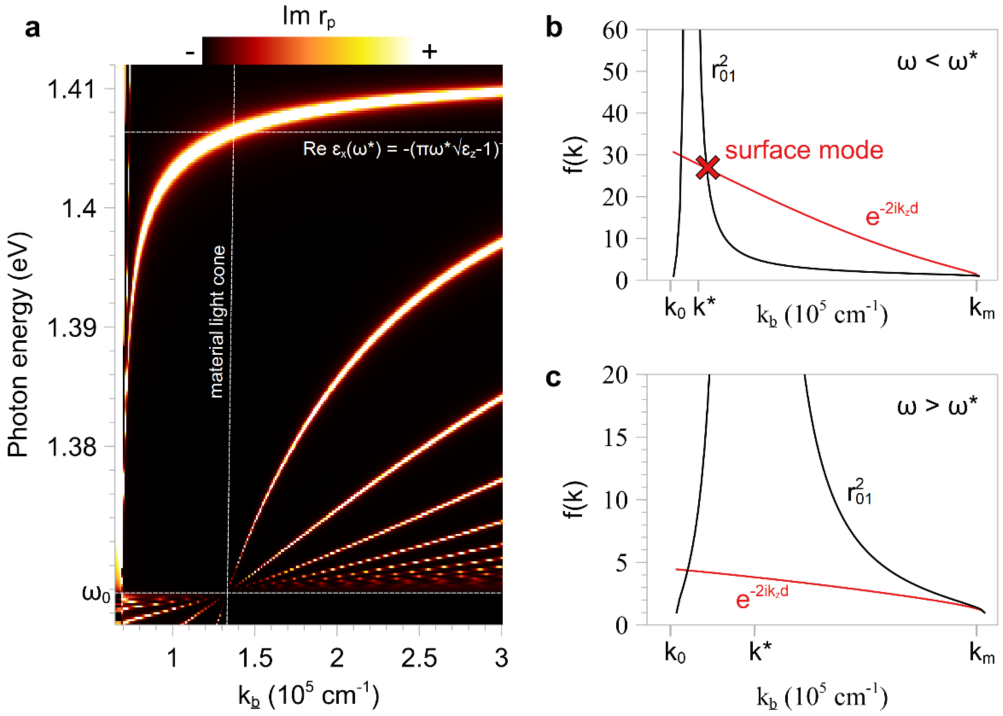

Supplementary Figure 5: Hyperbolic surface polaritons. **a**, hyperbolic polariton dispersion of a toy suspended anisotropic material with a single in-plane Lorentz oscillator dielectric function in the near-infrared. The fundamental mode is a surface mode with $k(\omega_0) \to k_0$ inside the material light cone and a bulk mode outside, crossing the material light cone at ω^* when Re $\varepsilon_x(\omega^*) = -\left(\pi\omega^* d\sqrt{\varepsilon_z - 1}\right)^{-1}$. Higher-order modes are always bulk modes with $k(\omega_0) \to k_m$. **b**, below ω^*, the two terms in Equation S10 intersect between k^* and k_m, corresponding to a surface mode solution. The initial slope of the exponential term is higher than that of r_{01}^2, but r_{01}^2 diverges at k^*. **c**, above ω^*, the two terms do not intersect above k^* because the initial slope of the exponential term is smaller.

Supplementary Note 2: Coupled excitons and waveguide modes

Coupling between excitons and light is often understood within the framework of the coupled oscillator model. In two-dimensional quantum well structures with mirrors, the self-oscillation condition for polaritons can be mapped analytically to a coupled oscillator model by making a near-resonance assumption[3]. In waveguide geometries, however, the self-oscillation condition cannot similarly be cast analytically into a coupled oscillator model and the exciton and "cavity mode" cannot be neatly decoupled into individual oscillators

A more general calculation for Rabi splitting energies involves computing the separation between complex-ω poles[4] of Equation S9. Complex-ω electrodynamics represent time-decaying electric fields[5], while our experiments probe spatially-decaying standing waves. Complex-ω electrodynamics do not represent our particular experiments, but are nonetheless physical and can be probed directly via other methods. Unlike complex-k solutions, complex-ω eigenvalues are homotopic to the coupled oscillator model and provide an unambiguous value for the Rabi splitting energy. In Supplementary Figure 6, we search complex-ω space for zeros of $\chi^2 = |1 - r_{01}r_{21}e^{2ik_zd}|^2$. To enforce zero-detuning, a real value for k can be chosen such that $\mathrm{Im}\,\omega_+ = \mathrm{Im}\,\omega_-$ when $\mathrm{Re}\,\omega_+ \neq \mathrm{Re}\,\omega_-$ (strong coupling); or $\mathrm{Re}\,\omega_+ = \mathrm{Re}\,\omega_-$ when $\mathrm{Im}\,\omega_+ \neq \mathrm{Im}\,\omega_-$ (weak coupling). If the dielectric function can be expressed as a single oscillator, then $\mathrm{Im}\,\omega_+ = \mathrm{Im}\,\omega_- = -\gamma/4$ at zero detuning in the strong coupling regime. We obtain Rabi splitting energies of $\Delta\omega = \omega_+ - \omega_- = 226\,\mathrm{meV}$ using the $295\,\mathrm{K}$ dielectric function and $d = 117\,\mathrm{nm}$, $k = 1.1222 \times 10^5\,\mathrm{cm}^{-1}$ (Supplementary Figure 6a) and $\Delta\omega = 163\,\mathrm{meV}$ using the $20\,\mathrm{K}$ dielectric function and $d = 107\,\mathrm{nm}$, $k = 1.05 \times 10^5\,\mathrm{cm}^{-1}$ (Supplementary Figure 6b). Reduced scattering rates at low temperature typically increase $\Delta\omega$, but other features of $\overleftrightarrow{\varepsilon}$ change simultaneously, particularly ε_z, leading to complicated temperature dependence. Finally, we plot complex-ω solutions for different detuning ($\mathrm{Re}\,k$) in Supplementary Figures 6c and 6d, showing that they coincide approximately with dispersions of split waveguide modes in the $\mathrm{Im}\,r_p$ loss function. Finally, we remark that the coupling strength is only a parameter of the coupled oscillator model and is not well-defined in the generalization described above.

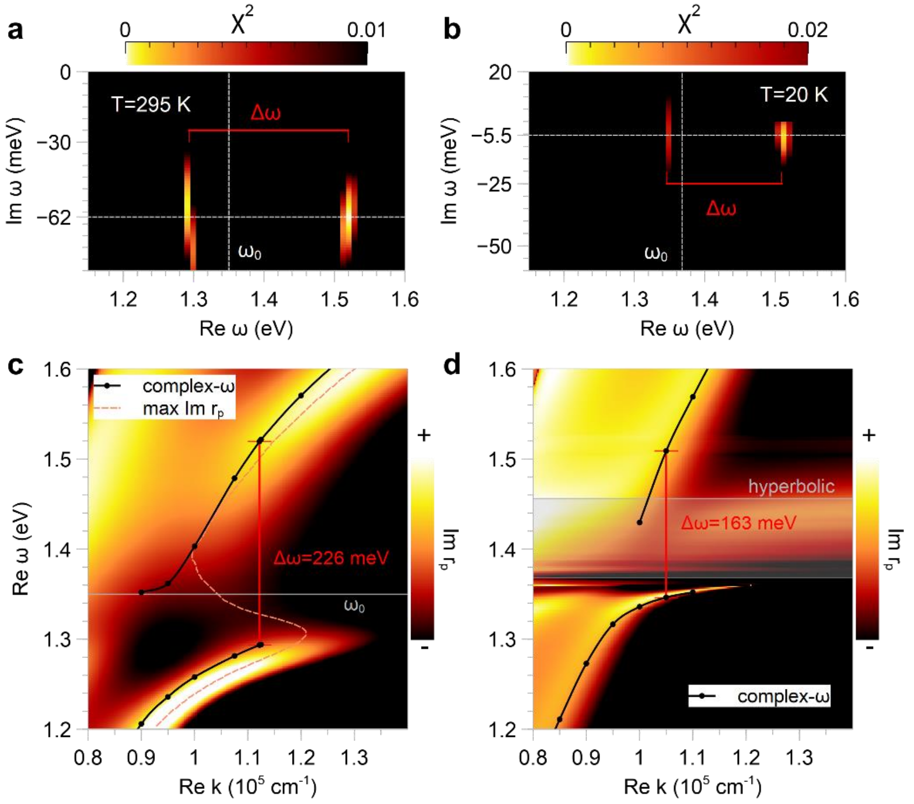

Supplementary Figure 6: Rabi splitting in complex-ω electrodynamics. Complex-ω poles of Equation S9 show Rabi splitting energies at zero detuning ($\mathrm{Im}\ \omega_+ = \mathrm{Im}\ \omega_-$) of **a**, 226 meV for Figure 2 sample at 295 K and, **b**, 163 meV for Figure 4 sample at 20 K. Complex-ω poles at different detuning approximately match split waveguide mode dispersions from, **c**, Figure 2 and, **d**, Figure 4 Im r_p loss functions.

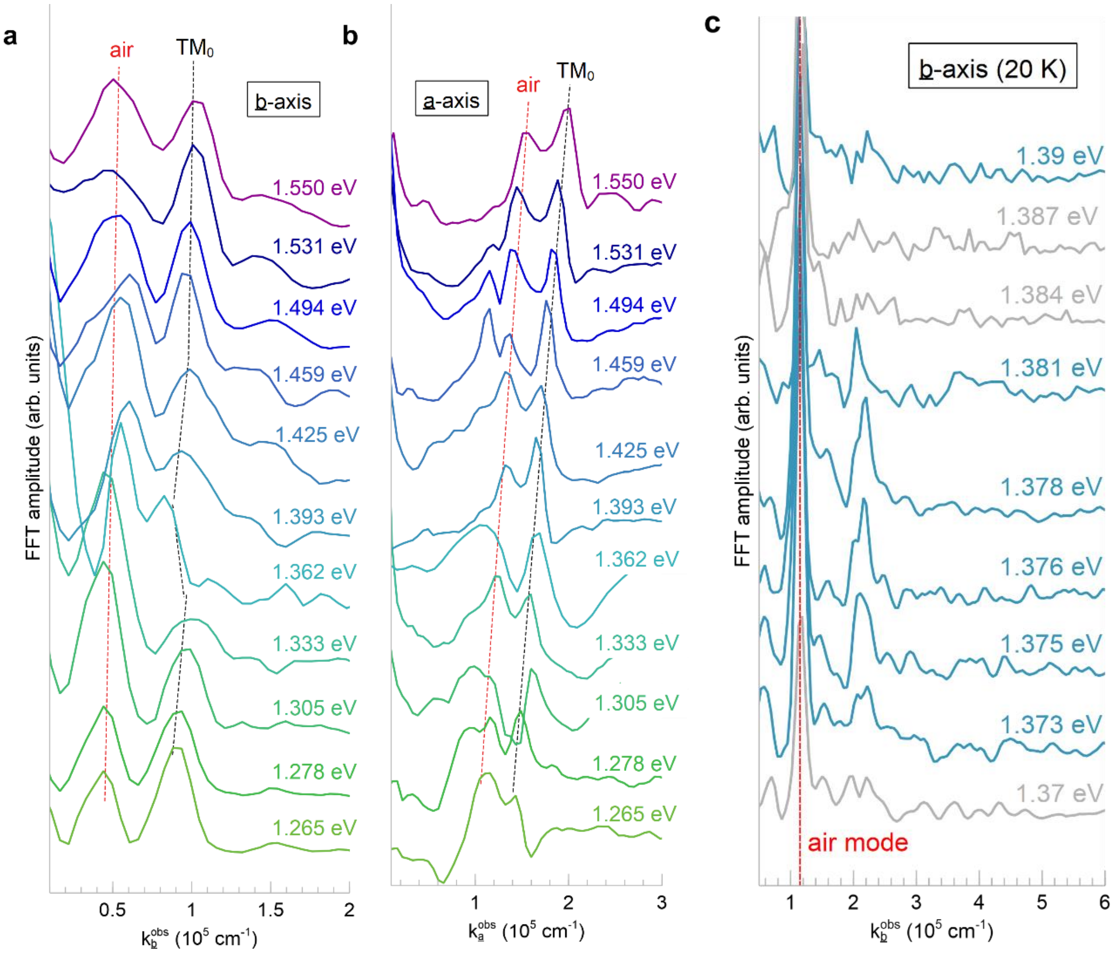

Supplementary Figure 7: Fourier analysis of near-field amplitude data. Fourier transforms of line profiles shown in, **a**, Figure 2c and, **b**, Figure 2d. Peak locations after geometrical correction correspond to plotted data points in Figure 2e. Red dashed line traces air mode peaks and black dashed line traces TM_0 waveguide mode peaks. A backbending dispersion is observed near the exciton energy (~1.35 eV) only along the $\underline{b}$-axis. **c**, Fourier transforms of $T = 20$ K near-field profiles. Blue peak positions are plotted in Figure 4c.

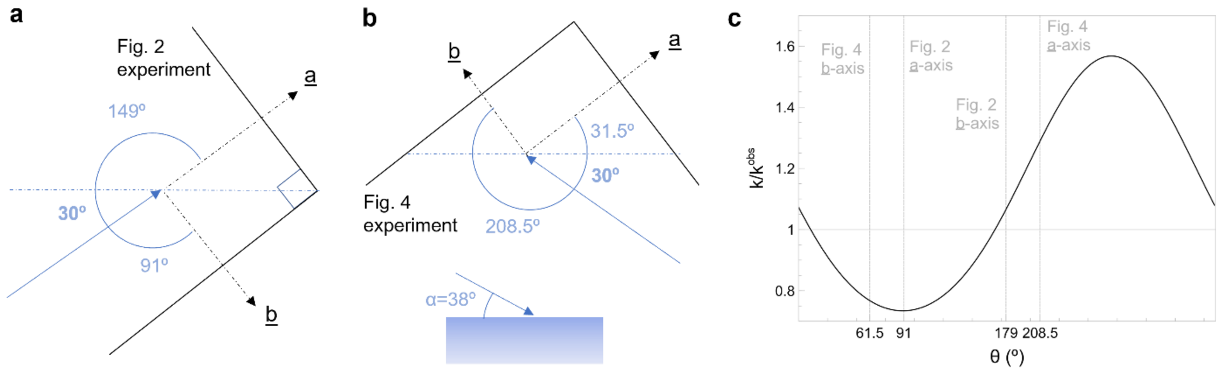

Supplementary Figure 8: Geometrical correction to the in-plane wavevector. a, schematic of sample geometry in the room-temperature near-field microscope (Figure 2 experiment) detailing the in-plane direction θ of incident light (blue arrow) relative to sample edges (solid black lines). **b**, similar schematic for Figure 4 sample in the cryogenic microscope. Inset shows light incident at an out-of-plane angle $\alpha = 38°$ for Figure 2 and 32.5° for Figure 4. Figure 3 sample configuration is $\theta = 90°,\ \alpha = 32.5°$. **c**, ratio of true wavevector to observed wavevector for different θ with $E = 1.376$ eV and $k = 1.67 \times 10^5\ \text{cm}^{-1}$. Relevant θ are marked with dashed gray lines.

Supplementary Note 3: Geometrical correction to the in-plane wavevector

Relative to mid-infrared polaritons, the in-plane wavevector of near-infrared exciton polaritons k is close to the momentum of free-space light $k_0 = \omega/c$. Modes launched by the s-SNOM tip thus tend to transmit through sample edges and outcouple to the far-field. Fringes in s-SNOM images are interferograms of edge-transmitted modes and light backscattered from the tip, which follow different paths back to the detector. Analysis of s-SNOM fringes must account for this path difference by applying a geometrical correction to extracted momenta[6]:

$$\frac{k}{k^{obs}} = \frac{k - k_0 \sin(\beta - \theta) \cos\alpha}{k \cos\beta} \tag{S14}$$

$$\beta \equiv \arcsin\left(\frac{k_0}{k} \cos\alpha \cos\theta\right) \tag{S15}$$

where k^{obs} is the observed momentum extracted from Fourier analysis, $\alpha = 38°$ is the out-of-plane incident angle[7], and θ is the in-plane incident angle relative to the sample edge parallel. Given k^{obs} and θ, one can solve Equations S14 and S15 self-consistently to obtain k. Supplementary Figures 8a and 8b detail θ for sample orientations used in Figures 2 and 4, respectively. Supplementary Figure 8c plots k/k^{obs} for fixed energy and true momentum.

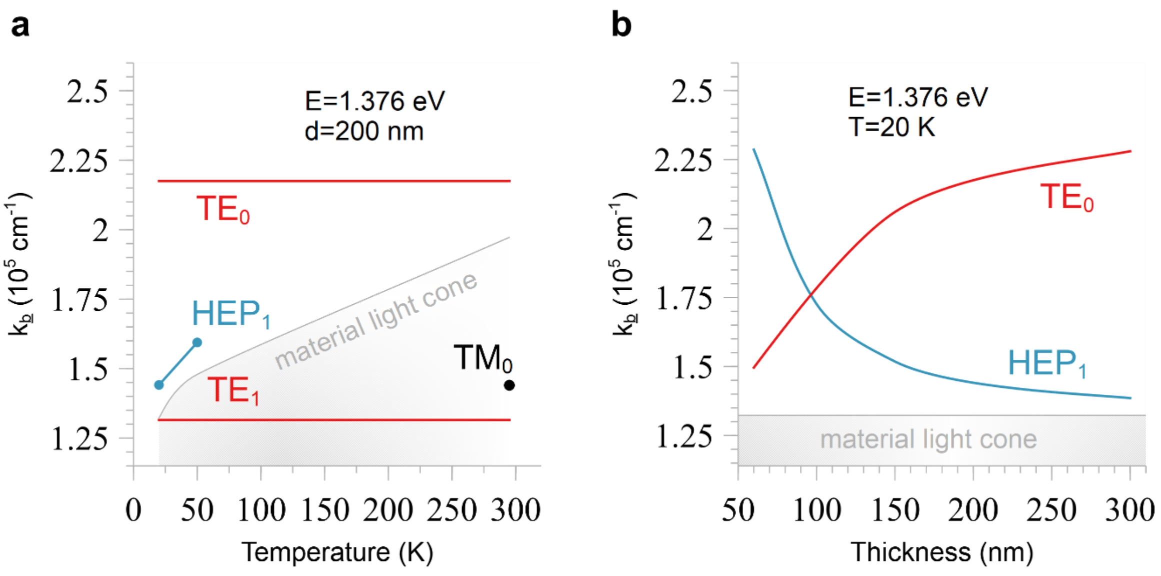

Supplementary Figure 9: Temperature and thickness dependence of hyperbolic exciton polaritons. Calculated, **a**, temperature and, **b**, thickness dependence of $n = 1$ hyperbolic exciton polariton (HEP_1) and fundamental transverse electric waveguide mode (TE_0) using far-field optical constants (assuming $\underline{a}$-axis is not temperature-dependent) and $\underline{c}$-axis dielectric constants from Supplementary Figure 3.

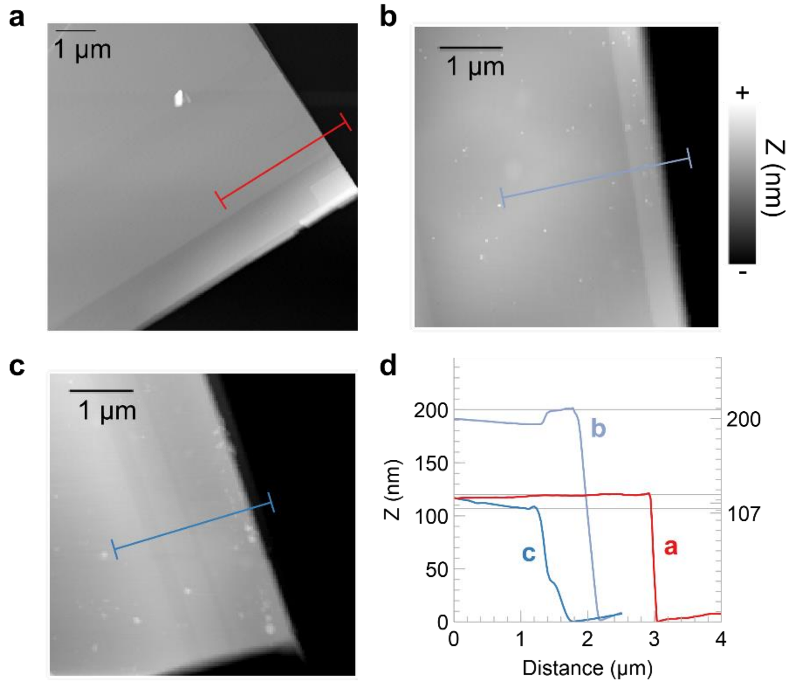

Supplementary Figure 10: Atomic force microscopy of CrSBr microcrystals. Atomic force microscopy topography images of CrSBr samples in, **a**, Figure 2, **b**, Figure 3 and, **c**, Figure 4. **d**, line profiles along the corresponding color-coded lines in topography images used to measure thicknesses of 117 nm, 200 nm, and 107 nm for samples in Figures 2, 3, and 4, accordingly.

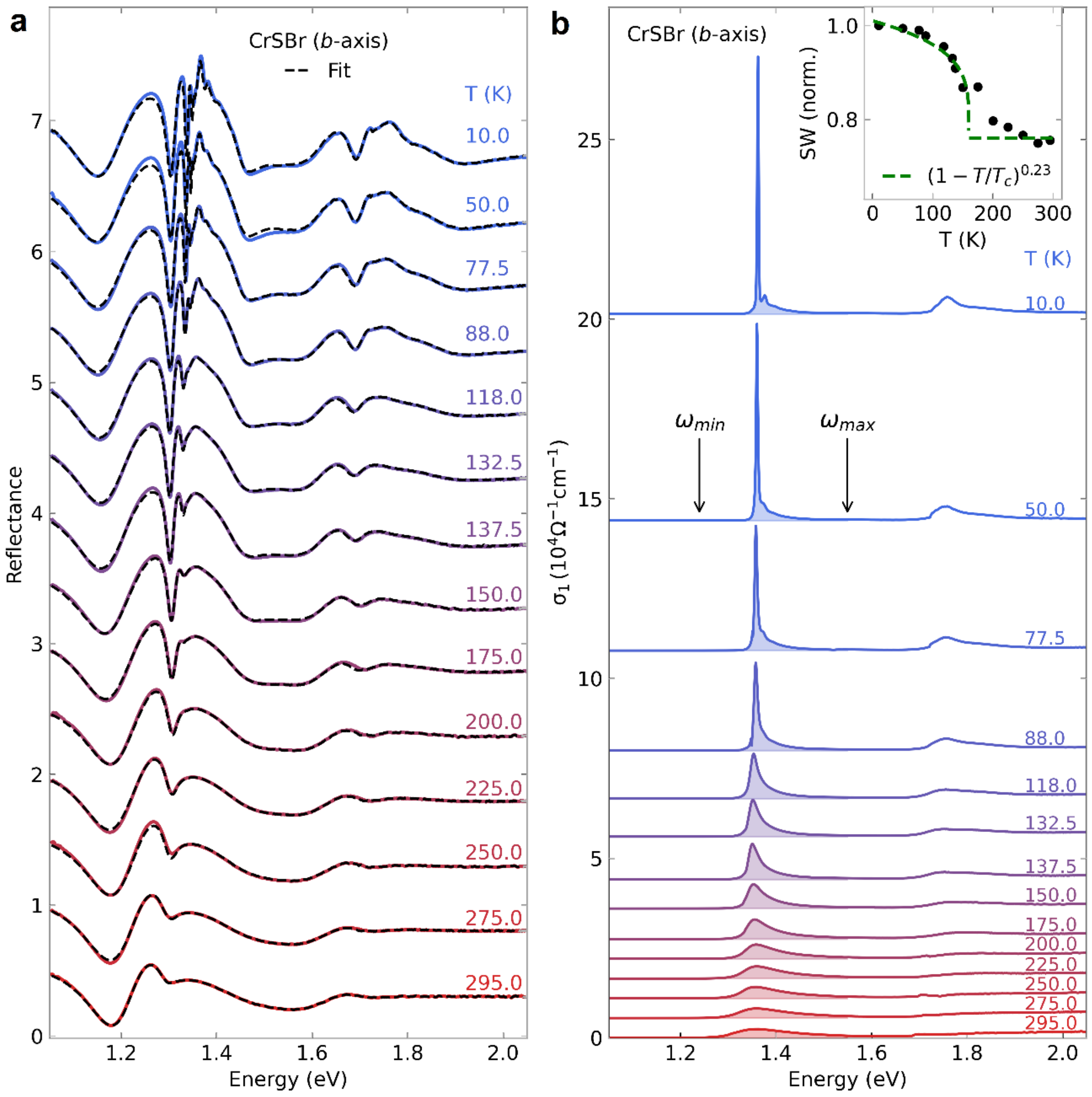

Supplementary Figure 11: Temperature dependence of exciton spectral weight. a, reflectance spectra polarized along the $\underline{b}$-axis of a bulk 297-nm-thick CrSBr microcrystal at temperatures $T = 10 - 295$ K. Variational dielectric function fits are black dashed lines. **b**, corresponding real optical conductivities σ_1. Filled areas correspond to areas of integration between ω_{min} and ω_{max} used to calculate the exciton spectral weight (*SW*). Inset shows *SW* as a function of temperature. Fitting a power law (green dashed line) yields a critical exponent of 0.23.

Supplementary References

[1] R.M.A. Azzam, N.M. Bashara, Ellipsometry and Polarized Light (North-Holland: New York, 1977)

[2] I. Abdulhalim, Analytic propagation matrix method for linear optics of arbitrary biaxial layered media, *Journal of Optics A: Pure and Applied Optics* **1**, 646-653 (1999)

[3] V. Savona, L.C. Andreani, P. Schwendimann, A. Quattropani, Quantum well excitons in semiconductor microcavities: unified treatment of weak and strong coupling regimes, *Solid State Communications* **93**, 733-739 (1995)

[4] A. Bylinkin, *et al*., Real-space observation of vibrational strong coupling between propagating phonon polaritons and organic molecules, *Nature Photonics* **15**, 197-202 (2021)

[5] A. Archambault, T.V. Teperik, F. Marquier, J.J. Greffet, Surface plasmon Fourier optics, *Physical Review B* **79**, 195414 (2009)

[6] A.J. Sternbach, *et al*., Femtosecond exciton dynamics in WSe_2 optical waveguides, *Nature Communications* **11**, 3567 (2020)

[7] D. Hu, *et al*., Probing optical anisotropy of nanometer-thin van der Waals microcrystals by near-field imaging, *Nature Communications* **8**, 1471 (2017)
